# Model-based inference of metastatic seeding rates in de novo metastatic breast cancer reveals the impact of secondary seeding and molecular subtype

**DOI:** 10.1038/s41598-022-12500-1

**Published:** 2022-06-08

**Authors:** Noemi Vitos, Philip Gerlee

**Affiliations:** 1 Bla Kustens Halsocentral, 57251 Oskarshamn, Sweden; 2 Mathematical Sciences, Chalmers University of Technology, 41296 Gothenburg, Sweden; 3 Mathematical Sciences, University of Gothenburg, 41296 Gothenburg, Sweden

**Keywords:** Breast cancer, Cancer models

## Abstract

We present a stochastic network model of metastasis spread for de novo metastatic breast cancer, composed of tumor to metastasis (primary seeding) and metastasis to metastasis spread (secondary seeding), parameterized using the SEER (Surveillance, Epidemiology, and End Results) database. The model provides a quantification of tumor cell dissemination rates between the tumor and metastasis sites. These rates were used to estimate the probability of developing a metastasis for untreated patients. The model was validated using tenfold cross-validation. We also investigated the effect of HER2 (Human Epidermal Growth Factor Receptor 2) status, estrogen receptor (ER) status and progesterone receptor (PR) status on the probability of metastatic spread. We found that dissemination rate through secondary seeding is up to 300 times higher than through primary seeding. Hormone receptor positivity promotes seeding to the bone and reduces seeding to the lungs and primary seeding to the liver, while HER2 expression increases dissemination to the bone, lungs and primary seeding to the liver. Secondary seeding from the lungs to the liver seems to be hormone receptor-independent, while that from the lungs to the brain appears HER2-independent.

## Introduction

The occurrence of distant metastasis for breast cancer is associated with a considerably worse prognosis, with a 5-year survival rate of 28%^1^. Increased knowledge about the extent of metastasis could guide clinicians in choosing effective therapies for different patients. Metastasis formation is a multi-step process which begins with the detachment of tumor cells from the primary tumor, making their way through the stroma to the bloodstream or lymphatic circulation, creating circulating tumor cells (CTCs)^2^. In the circulation, CTCs have to overcome challenges such as hemodynamic shear forces and the attack of the immune system, leading to a short survival time, estimated to be only several hours in breast cancer patients^3^. Formation of metastatic foci presents further challenges, and out of tens of thousands of tumorogenic cells injected into mice, only 100 were able to form metastatic foci^4^. This leads us to the conclusion that micrometastasis formation in downstream organs is necessary for metastasis formation. These micrometastases in turn shed CTCS in a process known as secondary seeding.

Mathematical models can lead to clinically valuable predictions by providing quantitative understanding of metastasis spread. For instance Benzekry et al. present a mechanistic model of metastasis in neuroblastoma that can describe clinical data and provide a computational biomarker with a predictive power of overall survival that is better than clinical data alone^5^. In contrast with the model mentioned above which are deterministic of nature, Newton et al. developed a stochastic model where the dissemination of cancer cells is modeled as an ensemble of random walkers on a network^6^. The idea of modelling metastasis spread on a network where links are routes of spread and nodes are organs was first described by Scott et al.^7^. Building on the model by Newton et al.^6^, Gerlee et al.^8^ took into account secondary seeding and presented a model that quantifies the rate of cancer cell dissemination between different organs. This model was applied to tongue and ovarian cancer and was able to make predictions in good agreement with clinical data^9^. In the present work we apply a similar framework to breast cancer, where we also model tumor growth in order to estimate tumor age. The model allows us to quantify dissemination rates between different organs and to predict the probability of developing bone, lung, liver and brain metastasis a certain time after tumor initiation for undiagnosed and untreated patients. This could aid clinicians in choosing more intense treatment options for patients with higher risk of developing certain types of metastasis.

## Methods

**Figure 1:**
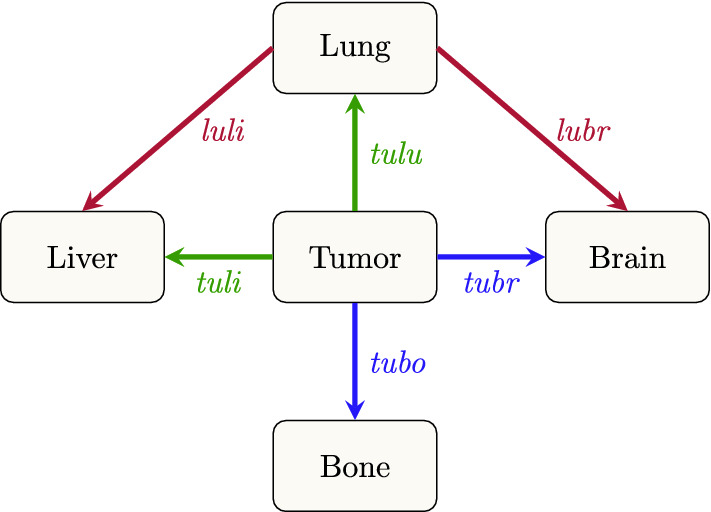
Seeding network. Seeding pattern between different metastasis sites and the primary tumor. Dissemination rate from tumor to bone represented by parameter “*tubo*”, tumor to lung by “*tulu*”, tumor to liver by “*tuli*”, tumor to brain by “*tubr*”, lung to liver by “*luli*” and lung to brain represented by “*lubr*”. Green arrows indicate primarily lymphatic spread, blue arrows venous spread and red arrows hematogenous spread.

We model a natural course of tumor development with a growing primary tumor without clinical interventions. As the SEER database used to parameterize the model only provides metastasis information at diagnosis, our model effectively represent the development of de novo metastatic breast cancer. We assume that dissemination rates between primary tumor and metastases do not change with time and are the same for various tumor types. Our model allows us to quantify the dissemination rate between tumor, bone, lungs liver and brain connected by a seeding network displayed in Fig. 1. The routes of metastasis formation for breast cancer have been debated and are not yet established^10^. The anatomical motivation behind our seeding network is presented in “Biological motivation behind seeding network” section, and in following sections we explain the likely routes to each metastatic location. We also motivate why axillary lymph nodes are not included (“Role of lymph nodes” section).

Combinations of different metastasis locations are referred to as states. These create a network with transition possibilities between states (see Fig. 4), representing evolution of a metastasis spread, with dynamics described in “Mathematical model development” section.

From the SEER database we extract information on patients tumor size and presence/absence of metastases in the lung, bone, liver, brain at diagnosis. The characteristics of the data used from the SEER database^11^ is presented in “Data” section. Finally, we will go through the mathematical framework behind the model in “Mathematical model development” section. When analyzing results we focus on how ER, PR and HER2 status influence dissemination rates, as these are the molecular properties recorded in the SEER database^11^. A tumor found positive for either ER or PR (ER+/PR−, ER−/PR+, ER+/PR+) is considered hormone receptor (HR) positive and a tumor negative for both ER and PR receptors is HR negative, giving rise to four possible subtypes: HR−/HER2+, HR+/HER2+, HR+/HER2− and HR−/HER2−.

### Biological motivation behind seeding network

**Figure 2:**
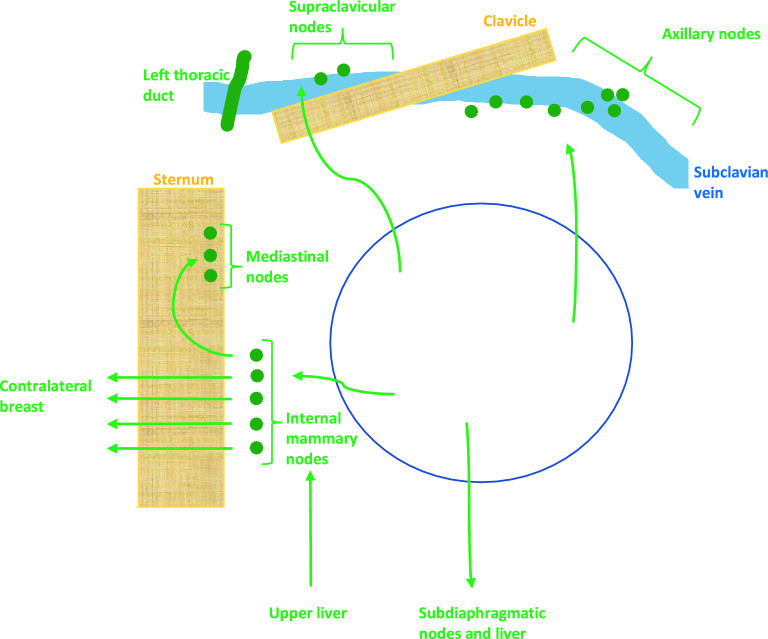
Lymphatic drainage of the breast. The lymph drainage of the breast takes three main routes: to axillary lymph nodes, internal mammary lymph nodes and less frequently directly to the supraclavicular nodes. The internal mammary nodes may receive drainage from the upper portions of the liver and deeper structures of the anterior abdominal wall. Lymph from these nodes can pass to the contralateral internal mammary nodes and mediastinal nodes. Lymphatics from the breast can also drain to subdiaphragmatic nodes and to the nodes of the liver (Gerota’s paramammary route).

In order to construct a network model which represents a biologically plausible seeding pattern, we investigate the anatomical possibilities. The lymph drainage of the breast takes three main routes: to axillary lymph nodes, internal mammary lymph nodes and less frequently directly to the supraclavicular nodes^12^, (see Fig. 2). The supraclavicular nodes drain the upper, superficial portions of the breast^13^. The axillary lymph nodes receive drainage from all quadrants of the breast in both the superficial and deep portions^14^. The internal mammary nodes too, receive lymph from all quadrants and drain the deep portions of the breast^14,15^. Lymph from these nodes can pass to the contralateral internal mammary nodes and mediastinal nodes^12^. The internal mammary nodes can also receive drainage from the upper portions of the liver and deeper structures of the anterior abdominal wall^16^. Finally, lymphatics from the breast can also drain to subdiaphragmatic nodes and to the nodes of the liver (Gerota’s paramammary route)^13^. Lymphatics from the left breast eventually drain into the thoracic duct and left subclavian vein, while those from the right breast drain into the right subclavian vein, both leading through the vena cava to the heart.

**Figure 3:**
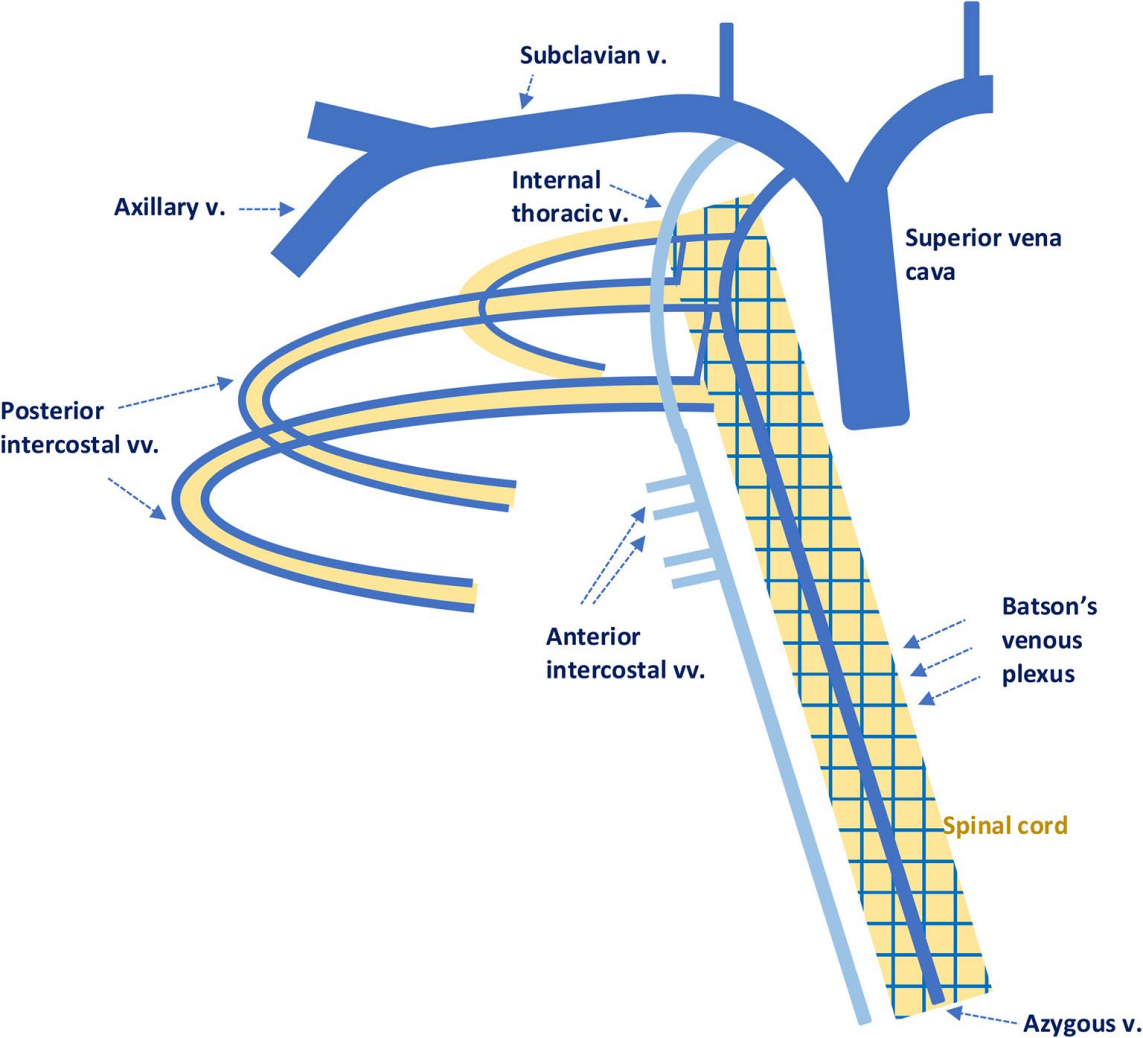
Venous drainage of the breast. The three major routes are to the internal thoracic vein, to the the posterior intercostal veins and to the axillary vein. Posterior intercostal veins drain into the azygous vein which communicates with Batson’s plexus displayed as a network of veins surrounding the spinal cord.

The venous drainage of the breast takes three major routes: to the internal thoracic vein, to the the posterior intercostal veins and to the axillary vein^17^, displayed in Fig. 3. All these veins eventually drain into the superior vena cava and through the heart the first capillary bed they encounter is in the lungs. Posterior intercostal veins drain initially into the azygous vein which communicates with valveless system of veins located in the epidural space called Batson’s vertebral venous plexus^18^. It regulates intracranial pressure with posture and drains the cerebral, abdominal and pelvic cavities^19^. Due to the lack of valves, an increased pressure in the vena cava system can result in backward flow of venous blood from the breast^20^, proposing a possible pathway for metastatic spread via Batson’s vertebral venous plexus to the vertebrae, skull, pelvis bone and the central nervous system.

### Lungs

Thomas et al.^21^ performed necropsies on 26 individuals who died in disseminated breast cancer and compared the frequencies with which intralymphatic and intravascular tumors were found in the lung, visceral pleura and parietal pleura. Ratios between intralymphatic and intravascular were 2.6:1 for lung parenchyma, 7:1 for visceral pleura and 11:1 for parietal pleura, implying that lymphatic spread is dominating. The most likely route of spread is from the ipsilateral internal mammary nodes by lymphatic communications to lymph node groups on both sides of the mediastinum and from there to the lung, pleura and mediastinum.

Genomic analysis and parsimonious reconstruction of the metastatic cascade of two patient cases by Cresswell et al.^22^ revealed seeding from the primary to the lung, from lung to the liver and from the liver to the ovary. El-Kebir et al.^23^ analyzed one of the same patient case and suggested a single-source monoclonal pattern of dissemination from the primary to the lungs and from the lungs to the liver, brain, rib and ovary. Both dissemination patterns suggest that there is a direct seeding route between the lungs and the breast.

Echeverria et al.^24^ used xenografts from triple-negative breast cancer patients with multiple metastasis in mouse models. They found that lung, liver, and brain metastases are enriched for an identical population of high-abundance subclones and share a genomic lineage. This suggests that either these three organs seed each other or that each one of them is seeded from the same primary tumor clone.

The above findings seem to support a direct seeding from the primary tumor to the lung, anatomically explained by spread from the internal mammary nodes via mediastinal lymph node groups to the lungs. Hematogenous spread from the veins draining the breast through the heart and to the lungs is also plausible.

### Liver

Stutte et al.^25^ examined 9 lymph node regions along the chest wall and the liver in 312 breast cancer patients sonographically. They found that liver metastases often occurred with internal mammary lymph node metastases, and that liver metastases were the only manifestation of distant metastasis in three patients with internal mammary lymph node metastases. This suggests that liver metastases of breast cancer can spread lymphatically from internal mammary lymph node metastases.

Thomas et al.^21^ investigated necropsies on 26 individuals who had died of disseminated breast carcinoma, and found that in a number of cases tumor metastasis were confined to the lower chest wall and diaphragm. These could be explained by lymphatic communications between the breast and liver via lymph nodes on the anterior surface of the diaphragm and lymphatic drainage of the upper surface of the liver to the internal mammary nodes. Backflow in these vessels could allow tumor cells to spread from the breast to the liver.

Genomic analysis by Cresswell et al.^22^ and El-Kebir et al.^23^ discussed in “Lungs” section support seeding from the lungs to the liver. We therefore model both a direct path between primary and the liver and a hematogenous spread via the lungs to the liver. The former represents lymph drainage of the breast to the liver as well as possible backflow in the lymphatics from the liver to the internal mammary nodes, under pathologic conditions.

### Bone

Hoadley et al.^10^ performed DNA whole genome and mRNA sequencing on primary tumors from two individuals with triple-negative/basal-like breast cancers. Their results suggested a direct seeding from the primary to the bone, while later analysis of the same patient data^23^ implied spread to the lungs and from there to the bone.

Venn diagrams of our patient data in Fig. S2 show that 54% of the lung metastasis patients have bone metastasis, while only 26% of the bone metastasis patients have lung metastasis. This simple comparison makes it seem unlikely that the spread would be from the lung to the bone. Hypothesizing that all cancer types make use of the same dissemination routes, we can compare the metastasis occurrence in breast and lung cancers. Wilson et al. found a similar incidence of bone metastasis in lung and breast cancer and the regional distribution of metastasis was the same^26^. According to Macedo et al. the relative incidence of bone metastasis in patients with advanced metastatic disease is 65–75% in breast cancer and 30–40% in lung cancer^27^. Thus, metastases to the bone are at least as frequent in breast cancer as in lung cancer.

The simplest explanation to the above presented statistics is that bone metastasis is not primarily seeded from the lung. Therefore we will have a separate direct dissemination paths from the primary to the bone, representing metastasis spread through the venous system, including Batson’s venous plexus^18^.

### Brain

Experiments on mice have shown that under pathological conditions, such as increased abdominal pressure, tumor cells can spread to the brain via the venous system^19^. This supports the hypothesis that Batson’s venous plexus serves as a venous channel connecting the cerebral, abdominal, and pelvic cavities.

According to Heitz et al. lung metastasis is the cancer type metastasizing most frequently to the brain indicating a functional pathway from the lungs to the brain^28^. As mentioned earlier, Echeverria et al. found that lung, liver, and brain metastases are enriched for an identical population of high-abundance subclones and share a genomic lineage^24^. This is further supported by the migration histories proposed by El-Kebir et al. based on genetic analysis^23^. In an autopsy study of central nervous system metastasis (including the dura matter and the brain) in breast cancer patients, it was found that the lungs seem to be seeding cancer spread^29^. In a later autopsy study they differentiated between the dura matter and the brain^30^. This study implied that dura matter metastases were seeded by the vertebral veins, while brain metastases were more likely to be seeded by lung metastasis.

Based on the above, we include two paths of dissemination to the brain; through homogeneous spread from the primary, via the lung to the brain or via a direct route through Batson’s venous plexus.

### Role of lymph nodes

Ullah et al.^31^ investigated the evolutionary history of metastatic breast caner in 20 patients and found that axillary lymph node metastasis was not involved in seeding the distant metastasis. Carter et al.^32^ investigated the relation of tumor size, lymph node status, and survival in 24,740 breast cancer cases using the SEER database, concluding that axillary lymph node status only serves as an indicator of the tumor’s ability to spread, rather than a central source of metastasis spread. Recent research also supports that axillary lymph nodes do not seed distant metastasis, but rather only have a prognostic value by reflecting the capability of cancer cells to metastasize^22,33^.

Above research suggests that the routinely tested axillary lymph nodes, presented in SEER database, do not have a key role in seeding metastasis, and thus we omit these from our model. Evidence for tumor seeding to the lungs and liver discussed above (“Lungs” and “Liver” sections), suggests that the internal mammary lymph nodes play a role in metastasis spread. In the USA however, no routine biopsy is done in these mammary glands, and thus no information is registered in the SEER database.

### Model overview

Based on the above anatomical motivations, we present a network displaying the allowed dissemination routes between tumor, bone, lung, liver and brain shown in Fig. 1. We assume a constant dissemination rate between organs; “*tubo*” (dissemination rate from tumor to bone), “*tulu*” (tumor to lung), “*tuli*” (tumor to liver), “*tubr*” (tumor to brain), “*luli*” (lung to liver) and “*lubr*” (lung to brain). These dissemination rate parameters represent the combination of; the rate of release of CTCs, their survival probability in the circulatory system and the probability of forming a metastasis in a downstream site.

**Figure 4:**
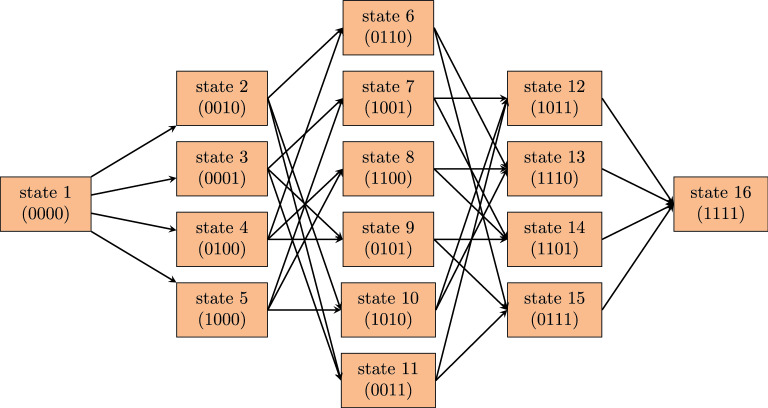
Network of states and transitions between them. Dissemination rates between states are represented by the six parameters *tubo* (tumor to bone), *tulu* (tumor to lung), *tuli* (tumor to liver), *tubr* (tumor to brain), *luli* (lung to liver), *lubr* (lung to brain), as follows: 1 $\rightarrow$ 2 *tuli*, 1 $\rightarrow$ 3 *tubr*, 1 $\rightarrow$ 4 *tulu*, 1 $\rightarrow$ 5 *tubo*, 2 $\rightarrow$ 6 *tulu*, 2 $\rightarrow$ 10 *tubo*, 2 $\rightarrow$ 11 *tubr*, 3 $\rightarrow$ 7 *tubo*, 3 $\rightarrow$ 9 *tulu*, 3 $\rightarrow$ 11 *tuli*, 4 $\rightarrow$ 6 *tuli* + *luli*, 4 $\rightarrow$ 8 *tubo*, 4 $\rightarrow$ 9 *tubr* + *lubr*, 5 $\rightarrow$ 7 *tubr*, 5 $\rightarrow$ 8 *tulu*, 5 $\rightarrow$ 10 *tuli*, 6 $\rightarrow$ 13 *tubo*, 6 $\rightarrow$ 15 *tubr* + *lubr*, 7 $\rightarrow$ 12 *tuli*, 7 $\rightarrow$ 14 *tulu*, 8 $\rightarrow$ 13, *tuli* + *luli*, 8 $\rightarrow$ 14 *tubr* + *lubr*, 9 $\rightarrow$ 14 *tubo*, 9 $\rightarrow$ 15 *tuli* + *luli*, 10 $\rightarrow$ 12 *tubr*, 10 $\rightarrow$ 13 *tuli* + *luli*, 11 $\rightarrow$ 12 *tubo*, 11 $\rightarrow$ 15, *tulu*, 12 $\rightarrow$ 16 *tulu*, 13 $\rightarrow$ 16 *tubr* + *lubr*, 14 $\rightarrow$ 16 *tuli* + *luli*, 15 $\rightarrow$ 16 *lubo*.

In our model, the four metastatic sites: bone, lung, liver and brain are represented as nodes which can take values 0 if no metastasis is present, and 1 if metastasis is present. Once a node has become positive it remains so, since metastasis are very unlikely to spontaneously regress. We refer to different metastatic combinations as states. Four nodes allows for $2^4$ states, however we only retain the 16 states allowed to form according to the seeding patterns in Fig. 1. We display all states and dissemination routes between them in Fig. 4. For example, having metastasis in the bone and the brain corresponds to state 7 (1001). One can arrive at this state in two different ways; direct dissemination from the tumor to the bone with dissemination rate ‘*tubo*’ and then from the tumor to the brain, with dissemination rate ‘*tubr*’ or in the reverse order.

In summary, we model a natural tumor development for de novo metastatic breast cancer with the following model assumptions:

- we take into account four metastatic locations; bone, lung, liver, brain and these are represented as nodes which can take values 1 or 0
- the dissemination rates between the primary and metastases are constant in time and the same for all molecular subtypes
- we assume a constant volume of metastases which implies a constant rate of secondary seeding
- the primary tumor has Gompertzian growth (see “Estimating time since tumor initiation” section) with no therapeutic interventions, and with parameters that are identical for all subtypes

### Data

We used data from the SEER*Stat case listing database Incidence—SEER 18 Regs Research Data + Hurricane Katrina Impacted Louisiana Cases, Nov 2017 Sub (1973–2015) with the following inclusion criteria: (I) female; (II) older than 18 years; (III) diagnosis confirmed by positive histology other than by other methods; (IV) breast cancer according to Site Recode ICD-O-3/WHO 2008 between 2010-2015; (V) belonging to 1 of the 4 subtypes: HR+/HER2−, HR+/HER2+, HR−/HER2+, and HR−/HER2−; and (VII) either positive or negative metastasis status at diagnosis in lung, bone, liver, brain; (VIII) histopathological information on tumor size (IX) maximum tumor diameter of 100 mm. A maximum tumor diameter of 100 mm ($\approx 10^{12}$ cells) was chosen, as this was estimated to be lethal tumor size by others^34,35^, and therefore 4010 patients were excluded.

In Table 1 we display the characteristics of the 317,166 patients that met our selection criteria with characteristics displayed in Table 1. Patient characteristics used are obtained at the time of diagnosis. For example, a patient with bone metastasis means: a patient that was found to have bone metastasis when diagnosed. For patients with multiple metastasis, we do not know what order they appeared from the data. All patients could be placed into one of the 16 states shown in Fig. 4, as all metastasis combinations are allowed. The majority of patients (98%) belong to state 1 (see Fig. S1). A comparison between the characteristics of subtype groups in our data selection and other published works is presented in Section S1.1.

**Table 1:** Data characteristics.

Characteristics	Subtype				
	All	HR−/HER2+	HR+/HER2+	HR+/HER2−	HR−/HER2−
No. of patients	317,166 (100)	13,406 (100)	32,504 (100)	235,828 (100)	35,428 (100)
**Diameter (mm)**					
$0 \le d < 20$	182,074 (57.4)	5562 (41.5)	15,239 (46.9)	146,431 (62.1)	14,842 (41.9)
$20 \le d < 40$	82,290 (25.9)	4352 (32.5)	10,291 (31.7)	55,281 (23.4)	12,366 (34.9)
$40 \le d < 60$	19,576 (6.2)	1387 (10.3)	2758 (8.5)	12,180 (5.2)	3251 (9.2)
$60 \le d < 80$	7073 (2.2)	551 (4.1)	936 (2.9)	4423 (1.9)	1163 (3.3)
$80 \le d < 100$	2755 (0.9)	233 (1.7)	372 (1.1)	1628 (0.7)	522 (1.5)
**Metastasis**					
Bone	7094 (2.2)	401 (3.0)	1164 (3.6)	4918 (2.1)	611 (1.7)
Lung	3109 (0.98)	334 (2.5)	536 (1.6)	1682 (0.71)	557 (1.6)
Liver	2649 (0.83)	407 (3.0)	650 (2.0)	1178 (0.5)	414 (1.1)
Brain	677 (0.21)	85 (0.63)	114 (0.35)	323 (0.14)	155 (0.44)
**Receptor status**					
HER2	45,910 (14.5)	13,406 (100)	32,504 (100)	0	0
ER	265,194 (83.6)	0	31,603 (97.2)	233,591 (99.1)	0
PR	231,177 (72.9)	0	23,881 (73.5)	207,296 (87.9)	0

Number of patients within each subtype group with a given characteristic specified in the left column. The numbers in parenthesis show the parentage of patients relative to respective subtype group.

### Mathematical model development

Every patient is assigned a tumor age (time since tumor initiation) and a state. Tumor age is estimated from tumor diameter by modelling tumor growth as described in “Estimating time since tumor initiation” section. The state of a patient is determined by their metastasis combination. The model assigns a probability to every patient, given their state and tumor age as described in “Calculating dissemination rates” section, for a range of different dissemination rate parameters. The likelihood of the whole data set is calculated for this range and dissemination rates corresponding to the highest likelihood are chosen.

### Estimating time since tumor initiation

Breast tumor growth has been investigated with mathematical models using both experimental and human data. The smallest detectable tumor size is around 2 mm in diameter ($\sim 10^7$ cells) and a lethal tumor volume is considered to be 100 mm in diameter $(10^{12}$ cells)^34,35^. We require a growth model that represents characteristics of tumor growth over this range and captures growth close to the maximum diameter; and therefore choose the Gompertz model^36^.

Tumor volume $V(t)$ governed by Gompertzian growth can be described at any time $t$ as

1 $$\begin{aligned} V(t)=V(0)e^{\frac{\alpha }{\beta }\left( 1-e^{-\beta t}\right) } \end{aligned}$$

where $V(0)$ is the volume of the tumor at initiation, time $t$ is time since initiation, $\alpha$ is the initial instantaneous growth rate, $\beta$ is the exponential rate of decrease of the growth rate. The Gompertz model has been shown to describe tumor growth in animal models well^37–39^. Norton et al. established a good fit of Gompertz model even on human breast cancer growth allowing for variability in $\beta$, resulting in a mean value of 0.0018 $\hbox {d}^{-1}$^40^.

The time it takes for a breast tumor to double in size was found to range between 105 and 270 days, with a weighted mean of 150 days^34^. The variation in different breast cancer subtypes measured by ultrasound yielded $103\pm 43$ days for triple-negative, $162\pm 60$ days for ER-positive and $241\pm 166$ days for HER2-positive tumors, respectively^41^. This is in line with the average of 150 days^34^.

Assuming the time it takes for a tumor to double in size is constant and is 150 days^34^, then the time it takes for a tumor to reach lethal size will be $16.4$ years. A Gompertz model with $\beta =0.0013$ $\hbox {d}^{-1}$ describes the system well. This value is in line with the mean value of $\beta = {0.0018}\,{\hbox {d}^{-1}}$ found by^40^. In order to obtain lethal tumor diameter of 100 mm and initial tumor size corresponding to a single cell with diameter 10 $\upmu \hbox {m}$, we choose $\alpha = 0.0359$ $\hbox {d}^{-1}$. Rearranging Eq. (1) we can calculate the time since tumor initiation (tumor age) for each patient, obtaining a distribution displayed in Fig. S3.

### Calculating dissemination rates

Transition rates between states described in Fig. 4 depend only on the present state of the system, so the network can be described as a continuous-time Markov chain. Only one of the nodes is allowed to become positive during a single transition. The transition rates between states depend on the sum of flow rates from all upstream sites (positive) and the sum of flow rates from downstream sites (negative) at that time. The master equation which describes the probability of being in state $i$ at time $t$ evolves according to:

2 $$\begin{aligned} \frac{dP_{i}(t)}{dt}=Q_{ii}P_{i}(t)+\sum _{j=1}^{16}Q_{ij}P_{j}(t) \end{aligned}$$

where time $t$ is the time that has passed since tumor initiation, $P_{i}$ is the probability of being in state $i$, $Q_{ii}$ is the rate of leaving state $i$, $Q_{ij}$ is the rate of moving from state $i$ to $j$ and the sum is over a total of 16 states displayed in Fig. 4. For example the probability of being in state 7 changes depending on the parameters *tubo*, *tubr* and *tuli*, *tulu* according to

3 $$\begin{aligned} \begin{aligned} \frac{dP_{7}(t)}{dt}&= Q_{77}P_{7}(t)+ Q_{73}P_{3}(t)+Q_{75} (P_{5}(t)) \\&=\left( -(tuli )-(tulu ) \right) P_{7}(t)+ (tubo )P_{3}(t)+(tubr )P_{5}(t). \end{aligned} \end{aligned}$$

We can rewrite Eq. (2) in matrix form

4 $$\begin{aligned} \frac{d{\mathbf {P}}(t)}{dt}=Q{\mathbf {P}} \end{aligned}$$

where ${\mathbf {P}}(t)= \left[ P_1(t), P_2(t), P_3(t), \dots \right]$ is a vector of probabilities and $Q$ is the transition matrix. Assuming that at tumor initiation the patient has only a primary tumor and no metastasis; i.e. $P_{1}(0)=1$ and $P_{i}(0)=0$ for $i>0$ , we can solve the equation numerically using the Euler forward method. We obtain a matrix $\widehat{P}$ where the elements in a column represent the probability of being in a certain state at time $t$ and there is one time step $\Delta t$ between neighbouring columns.

5 $$\begin{aligned} \widehat{P}= \begin{bmatrix} P_1 \left( 0, \theta \right) &\quad \dots &\quad P_1 \left( t, \theta \right) &\quad P_1 \left( t+\Delta t, \theta \right) &\quad \dots \\ P_2 \left( 0, \theta \right) &\quad \dots &\quad P_2 \left( t, \theta \right) &\quad P_2 \left( t+\Delta t, \theta \right) &\quad \dots \\ P_3 \left( 0, \theta \right) &\quad \dots &\quad P_3 \left( t, \theta \right) &\quad P_3 \left( t+\Delta t, \theta \right) &\quad \dots \\ . &\quad \dots &\quad . &\quad . &\quad . \\ . &\quad \dots &\quad . &\quad . &\quad . \\ . &\quad \dots &\quad . &\quad . &\quad . \\ \end{bmatrix} \quad \end{aligned}$$

$\theta$ represents the vector of dissemination rate parameters $\theta = \{ tubo , tulu , tuli , tubr , luli , lubr \}$.

We use the maximum likelihood method to estimate the parameters. We can express the likelihood of the data set as

6 $$\begin{aligned} L(\theta )=\prod _{j=1}^{16} P_{s_j} \left( t_j ,\theta \right) \end{aligned}$$

where $s_j$ is the state of patient $j$ and $t_j$ is the time since initiation until diagnosis for patient $j$. We minimize the logarithm of the negative of the likelihood function using the MATLAB function fminsearchbnd, and thereby obtain the most likely set of parameters given the data. We obtain these optimal parameters for the whole data set and also for each subtype group separately. For each parameter set we calculate the confidence intervals (CI) using parametric bootstraping^42^ with 50 samples, see Section S1.2.

## Results

### Dissemination rates

**Figure 5:**
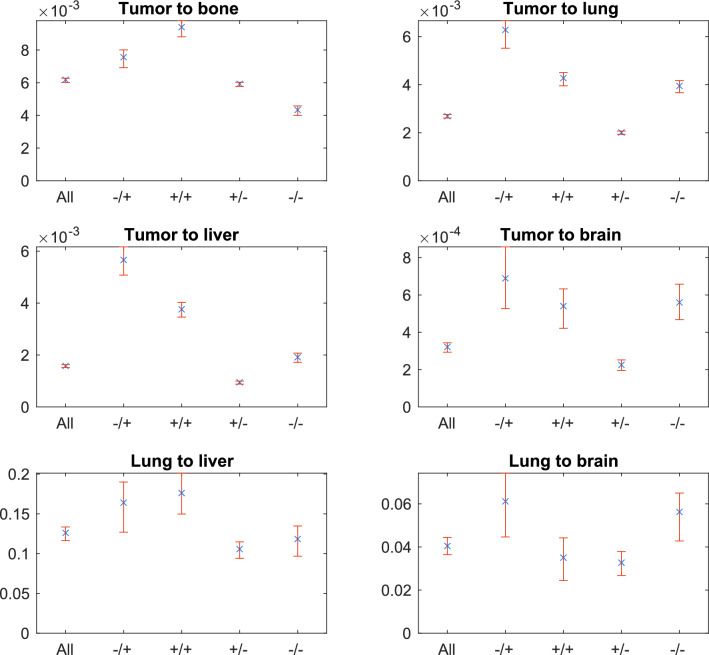
Dissemination rates and their 95% confidence intervals for each parameter. ‘All’ stands for the whole data set, while ‘−/+’ indicates HR−/HER2+, ‘+/+’ indicates HR+/HER2+, ‘+/−’ indicaates HR+/HER2−, ‘−/−’ indicates HR−/HER2− subtypes.

In Fig. 5 we present the dissemination rates when parameterizing using the whole data set as well as different subtype groups. Numerical values are displayed in Table S1.

### Model validation

**Figure 6:**
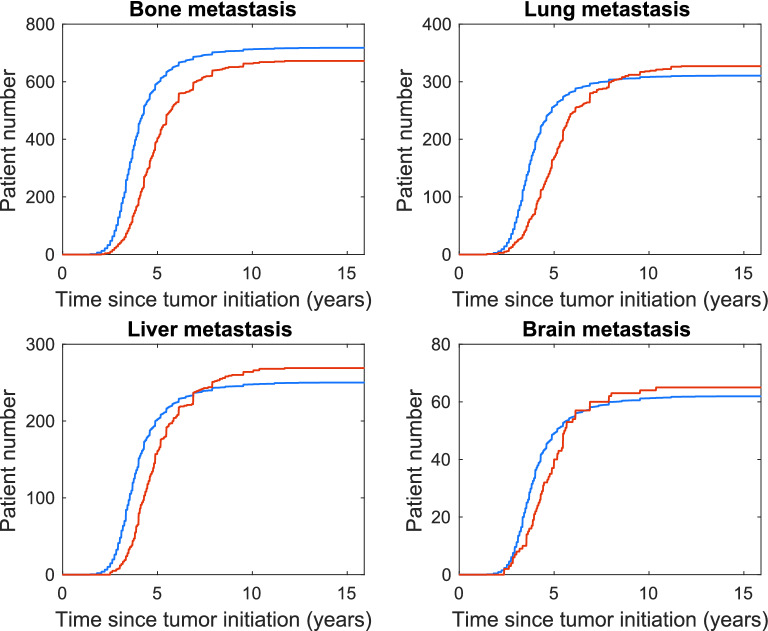
Model and one of the validation data sets using tenfold cross-validation. Cumulative number of patients versus tumor age. Data displayed in red and model prediction in blue.

We use tenfold cross-validation to validate our model by first randomly dividing our data into 10 equal groups, with $D$ number of patients in each group. Excluding one of the groups, referred to as the validation group, we use the remainder of the data to obtain model parameters. We use these parameters in our model to predict the cumulative number of patients within the validation group, with bone, lung, liver or brain metastasis at a tumor age $t_k$ or younger. Calculations on cumulative number of patients are described in Section S1.3. We repeat this procedure, excluding one of the 10 groups each time, comparing model predictions with the validation group by calculating the mean absolute percentage error (MAPE):

7 $$\begin{aligned} {\text {MAPE}}=\frac{100}{M} \sum _{t_k=0}^{t_k=T} \bigg | \frac{ x^{\text {data}}_{t_k}-x^{\text {model}}_{t_k} }{x^{\text {model}}_{t_k}}{\bigg |} \end{aligned}$$

where *M* is number of data points, ${t_k}$ is time at the beginning of time increment *k*, *T* is the maximum tumor age, $x^{\text {data}}$ is patient number according to data and $x^{\text {model}}$ is patient number predicted by model. We then take the average MAPE for the 10 cases. This is done for the whole data set as well as each subtype group with average MAPE values summarized in Table S2. One of these 10 comparisons for the whole data set is displayed in Fig. 6.

### Probability of developing metastasis

The model makes it possible to estimate the probability of developing a certain metastatic combination for patients who have not received any treatment, a certain time after tumor initiation. We assumed that a tumor of a given diameter represents an average tumor a certain time after tumor initiation and then used Gompertzian growth model to convert from tumor size to tumor age. For instance, $P_{12}(2)$ gives us the probability of developing a metastasis in the lung, liver and brain within 2 years since tumor initiation. Adding up all metastasis containing states we obtain the probability of developing any metastasis within a certain time (Fig. S4).

## Discussion

We have parameterized a continuous-time Markov chain network model that describes the formation of metastases with the help of patient data from the SEER register, thereby quantifying the relative tumor cell dissemination rates between metastasis stations. We investigated how the dissemination rates changed in different subtype groups and metastasis types. Using these values, the model can predict the probability of developing a metastasis a certain time after estimated tumor initiation for untreated patients.

### Model error

As displayed in Table S2 the best match seems to be for brain metastasis, while worst is for liver metastasis. The model is best at describing the HR−/HER2− subtype and worst at describing the HR+/HER2+ subtype group.

### Dissemination rates

Estimated tumor cell dissemination rates reflect the rate of release of circulating tumor cells, their survival probability and ability to form metastasis at a downstream site. The parameters representing dissemination rates from the tumor (*tubo*, *tulu*, *tuli*, *tubr*) are 5–300 fold smaller than the two parameters representing dissemination from the lung (*luli*, *lubr*), displayed in Table S1. This may be due to the fact that secondary seeding is much more effective in spreading tumor cells^43^. This could biologically be explained by metastatic cells being superior to primary tumor cells, as they have evolved from cells that have developed characteristics such as stress tolerance within the vasculature, anchoring to the vasculature wall and adaptation to a new microenvironment.

The relative size of the dissemination parameters to each other is preserved throughout subtypes, in descending order: *luli*, *lubr*, *tubo*, *tulu*, *tuli* and *tubr*, which means that the relative importance of dissemination routes in the different subtype groups is similar.

We investigated the effect of of HER2− and HR-positivity on the dissemination parameters. Hormone receptor positivity increases dissemination to the bone (comparing rates in the HR−/HER2− vs. HR+/HER2− and HR−/HER2+ vs. HR+/HER2+, see Fig. 5), while decreasing spread to the lung and direct spread to the liver. Both direct and indirect spread to the brain seems to be decreased by hormone receptor positivity, except in the HER2-positive case of direct dissemination, which seems to be hormone receptor-independent. Spread from the lung to the liver also seems to be hormone receptor-independent.

HER2-positivity promotes dissemination rate to the bone, lungs and direct dissemination to the liver. It increases direct dissemination to the brain and dissemination form the lung to the liver in the HR-positive cases, and does not seem to influence the HR-negative cases. Metastasis spread from the lung to the brain seem to be HER2-independent.

To the liver and the brain we have both primary (from the tumor) and secondary (from the lung) dissemination. By dividing *luli* with *tuli* and *lubr* with *tubr*, we find the relative importance of secondary and primary dissemination in different subtype groups, (see Table S3). We found that secondary dissemination from the lung to the liver is most important in the HR+/HER2− subtype group and least important in the HR−/HER2+ group. In the case of the brain secondary seeding seems to be significantly more important for HR+/HER2− subtype than the HR+/HER2+.

Finally, we compare our results to other network models of metastasis spread. Newton et al. created a similar Markov chain model on lung cancer with dissemination possibilities to 27 different organs^6^. They found the ratio of transition probability from lung to liver and lung to brain to be 0.284 and 0.565 respectively, which is in agreement with the ratios obtained from our model 0.314 and 0.332, even though dissemination is from different primary tumors. We can also compare incoming and outgoing dissemination rates for the lungs. The dissemination rate into the lung (0.00269) is much smaller than the total dissemination out of the lung (0.126 + 0.0404). This makes the lungs a good secondary seeding site, which is in line with a later model of Newton et al.^44^.

### Colonizing ability

The dissemination route from the lungs to the liver and the brain can anatomically be explained by hematogenous spread. The relative blood flow to these organs is estimated to be 6.5% and 12% of the cardiac output^45^. By dividing the relative dissemination rates we can get an estimate of the relative colonizing ability of breast cancer cells in the liver versus brain. Review data from 3827 autopsies was analyzed by gross examination and hematological sections to analyze the metastatic pattern of 41 different primary cancers and 30 different metastatic sites^9^. The ratio of metastasis number in liver versus brain for breast cancer was 5.2:1, while we find a ratio for relative metastasis forming ability to be 5.6 (95% CI 5.5–5.9), which is reasonably close. Breast cancer cells are thus more than fivefold better at colonizing the liver environment compared to the brain.

### Clinical significance

We estimated the probability of developing metastasis in any combination of the four organs (Fig. S4). Our model estimates the age of a 5 mm, 10 mm, 40 mm tumor to be 2.4, 2.9 and 4.9 years respectively. The probability of developing a metastasis within 10 years without any intervention is given by the probabilities corresponding to a tumor aged 12.4, 12.9 and 14.9 years: 12.5% (95% CI 12.1–12.7), 13.2% (95% CI 12.6–13.3) and 14.8% (95% CI 14.4–15.1) respectively.

We can compare this with the probability of developing relapsed metastatic disease after surgery for primary tumors of the same size. A study of 4797 patients investigated the probability of metastasis development within 10 years after intervention and found a linear relationship between tumor size at intervention and the probability of metastasis development^46^. For a 5 mm, 10 mm, 50 mm tumor they found a probability of approximately 10%, 15% and 50% respectively^46^. The discrepancy between these numbers and those obtained from our model are due to the fact that we model untreated patients whereas the patients that experience relapsed metastatic disease have been subject to surgery. This may be due to the fact that de novo and relapsed metastatic disease are biologically different, as suggested by a recent study^47^.

Even though the model is restricted to de novo breast cancer it could become clinically significant when it comes to screening for metastasis at diagnosis. When the model has been validated on independent data it could inform clinicians when they make decisions on which patients to screen for distant metastases.

An important future improvement to our model in order to make it clinically more useful is to parameterize it with data obtained post-resection, in which case it could be used in order to focus monitoring of recurrent disease rates. A further improvement would be to account for subtype specific variation in tumor growth.

## Conclusions

Our model gives valuable insights into the relative importance of metastatic dissemination routes between organs. Seeding of tumor cells from metastases seems to be several hundred fold more important than seeding from the primary. Hormone receptor positivity enhances dissemination to the bone and diminishes dissemination to lungs and direct dissemination to the liver, while HER2 promotes dissemination the bone, lungs and direct dissemination to the liver. Secondary seeding from the lungs to the liver seems to be hormone receptor-independent, while spread from the lungs to the brain appears HER2-independent. The model also allows us to quantify metastasis formation ability in the liver and the brain, suggesting that breast cancer cells are several times better at colonizing the liver than the brain. Once validated on independent data the model could be used to guide screening for metastasis at diagnosis. Important further developments to the model include growth dynamics of metastases and allowing for changing dissemination rates with time.

## Supplementary Information

Supplementary Information.
