# Supplementary material for: Model-based inference of metastatic seeding rates in de novo metastatic breast cancer reveals the impact of secondary seeding and molecular subtype
Source: Sci Rep. 2022 Jun 8;12:9455. doi: 10.1038/s41598-022-12500-1 (PMC9177582; doi:10.1038/s41598-022-12500-1)
Supplement: Supplementary file 1 — Supplementary Information. [file 41598_2022_12500_MOESM1_ESM.pdf]

Supplementary Material

S1 Supplementary Methods

S1.1 Data selection and inclusion criteria

We used data from the *SEER*Stat case listing database Incidence - SEER 18 Regs Research Data + Hurricane Katrina Impacted Louisiana Cases, Nov 2017 Sub (1973-2015).* The primary cohort of patients consisted of breast cancer cases according to Site Recode ICD-O-3/WHO 2008 between 2010-2015. The inclusion criteria was the following: (I) female; (II) older than 18 years; (III) diagnosis confirmed by positive histology other than by other methods; (IV) breast cancer according to Site Recode ICD-O-3/WHO 2008 between 2010-2015; (V) belonging to 1 of the 4 subtypes: HoR+/HER2-, HoR+/HER2+, HoR-/HER2+, and HoR-/HER2-; and (VII) either positive or negative metastasis status at diagnosis in lung, bone, liver, brain; (VIII) histopathological information on tumor size (IX) maximum tumor diameter of 100 mm. A maximum tumor diameter of 100 mm ($\approx 10^{12}$ cells) was chosen, as this was estimated to be lethal tumor size by others [S50], and therefore 4010 patients were excluded.

S1.2 Data selection compared to published data

We can compare our data selection to previously published statistics on breast cancer subtypes. Subtype groups in the literature; luminal A, luminal B, basal-like do not correspond completely to our subdivisions as we did not have access to all necessary molecular data. We will assume that a sufficient similarity between our HoR+/HER2-, HoR-/HER2+ and the luminal subtypes and similarly between our HoR-/HER2- subtype and the basal subtype. Triple-negative corresponds to our HoR-/HER2-, and HER2-enriched corresponds to HoR-/HER2+.

The HoR-/HER2- subtype has less bone metastasis compared to other subtypes, which is in line with analyses of others of the basal subtype [S51]. HoR+/HER2+ and HoR-/HER2+ had higher occurrence of lung, liver and brain metastasis compared to HoR+/HER2- in line with the findings of Kennecke et al. [S52]. We found that HoR-/HER2+ subtype is associated with highest incidence of liver metastasis, also found by Wei et al. [S53] who concluded that HER2 subtype demonstrated a significant liver-homing characteristic compared to the luminal and triple-negative subtypes. HoR-/HER2- and HoR-/HER2+ subtypes have highest probability of developing brain metastasis compared to the other two subtypes, also concluded by Heitz et al. [S54] and Largillier et al. [S55].

S1.3 Initial guess values and confidence intervals

The ideal parameter values may vary depending on the initial guess of the optimization algorithm. We use a range of initial guess parameters which give probability values ranging from 0 to 1, and choose the set of parameters which gives the maximal likelihood. Each ideal parameter set was used 50 times to generate synthetic data, with the same number of patients as in our original data set. We use this synthetic data set to obtain new maximum likelihood estimates of the dissemination parameters. The confidence intervals are calculated as the 5% and 95% quantiles of these 50 values.

S1.4 Model predictions versus data

We calculate model predictions by dividing our patient cohort with D number of patients, into time increments Δt; with N_k number of patients in increment $(t_k - \Delta t) < t < t_k$, where t_k is time at the beginning of time increment k.

We calculate distribution of patients between the 16 states at time t_k as

$$n_i(t_k) = N_k P_i(t_k) \tag{S1}$$

where $n_i(t_k)$ is the predicted number of patients in our cohort in state i in the time interval $(t_k - \Delta t) < t < t_k$ and $P_i(t_k)$ is the probability of being in state i at t_k.

The predicted cumulative number of patients in a certain state i with tumor age t_k or less is obtained by

$$n_i(t < t_k) = \sum_{t=t_1}^{t=t_k} n_i(t) \tag{S2}$$

For calculating the cumulative number of patients with for example lung metastasis, we sum over all states containing lung metastasis.

The corresponding cumulative patient number given by the data is obtained by dividing the number of patients with a certain metastasis type and tumor age $t \leq t_k$ by the total number of patients in our data set D.

S2 Supplementary Figures

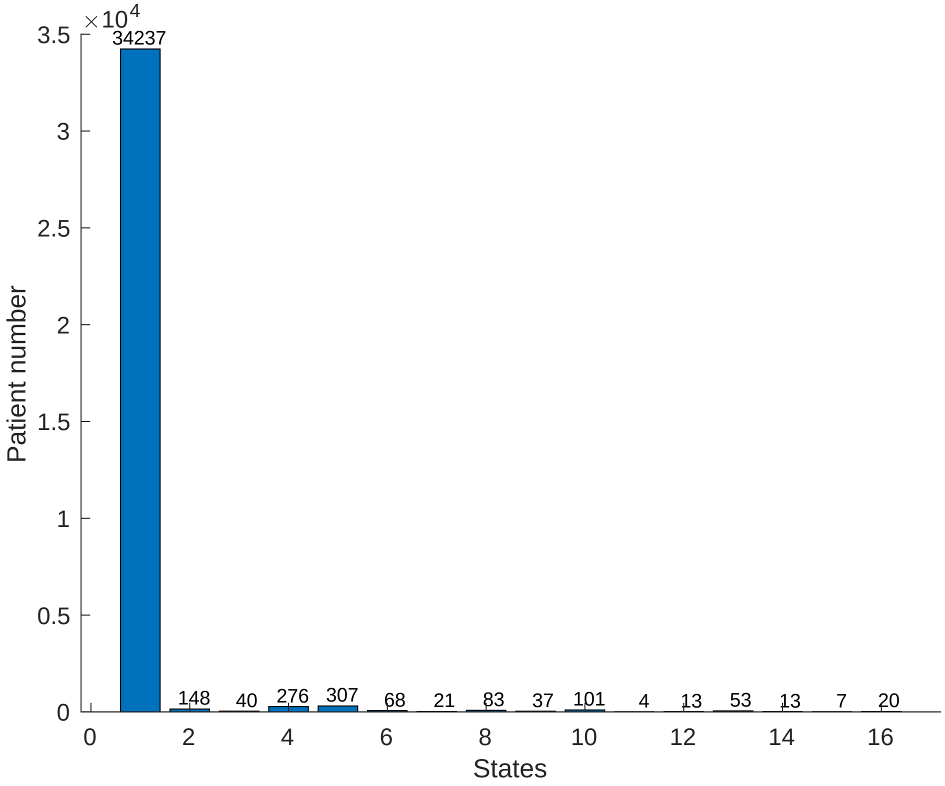

Figure S1: Distribution of patient cohort amongst 16 states. Numbers above bars correspond to number of patients in that state.

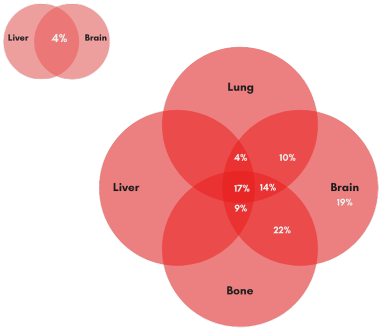

(a) Percentages with respect to total number of patients with brain metastasis.

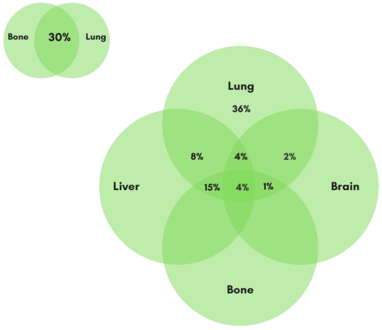

(b) Percentages with respect to total number of patients with lung metastasis.

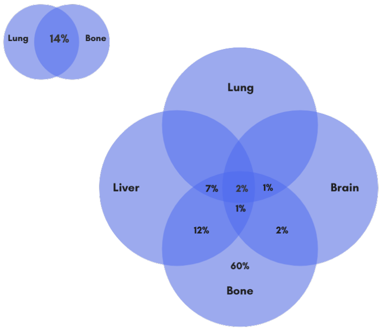

(c) Percentages with respect to total number of patients with bone metastasis.

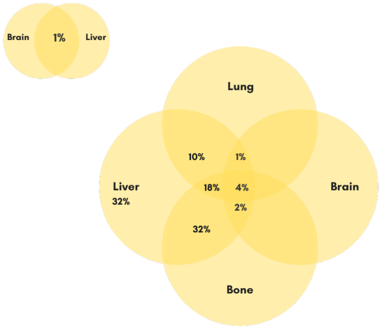

(d) Percentages with respect to total number of patients with liver metastasis.

Figure S2: Venn diagram displaying the metastasis variation as a percentage of certain metastasis type. Percentages with respect to total number of patients with metastases in respective organ. Numbers inside circles reflect number of patients with metastasis only in the organs in the corresponding circle areas. For example 60% of patients with bone metastasis had metastasis only in the bone as displayed in (c). 15% of patients with lung metastasis also had metastases in the liver and bone as shown in (b).

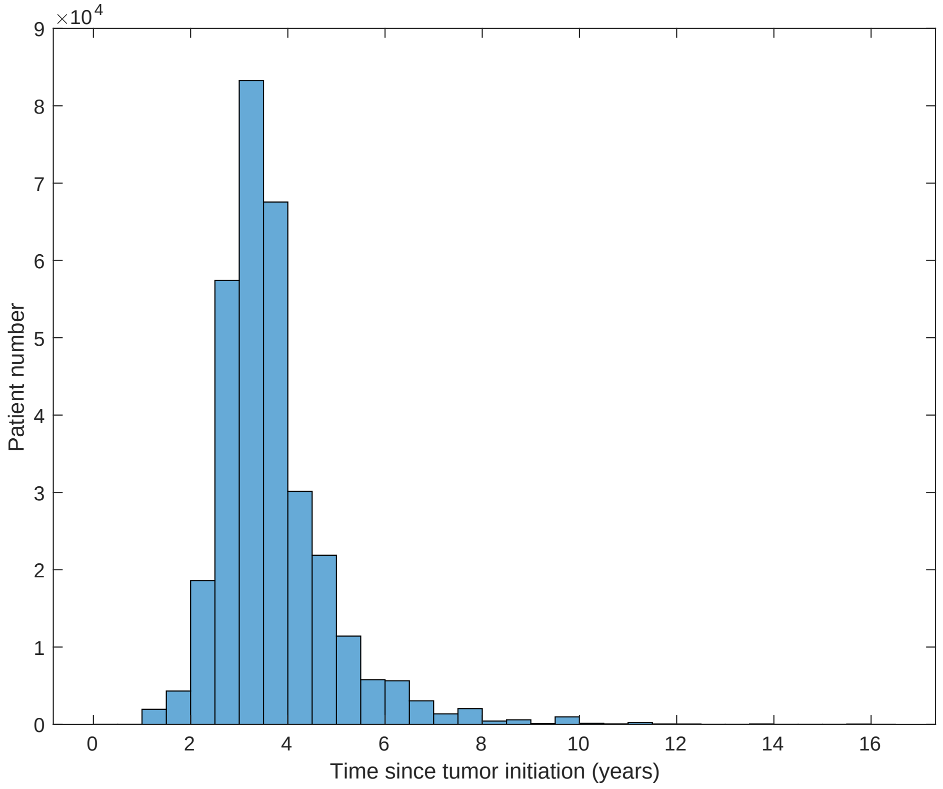

Figure S3: Distribution of patients since tumor initiation using Gompertz model. Growth rate parameter $\alpha = 0.0359\ \mathrm{d}^{-1}$ and $\beta = 0.0013\ \mathrm{d}^{-1}$, yielding a maximum tumor volume of $V_{max} = 5.2 \cdot 10^5\ \mathrm{mm}^3$ corresponding to lethal tumor diameter of 100 mm. Initial tumor size $5.2 \times 10^{-7}\,\mathrm{mm}^3$ corresponding to a single cell with diameter 10 µm.

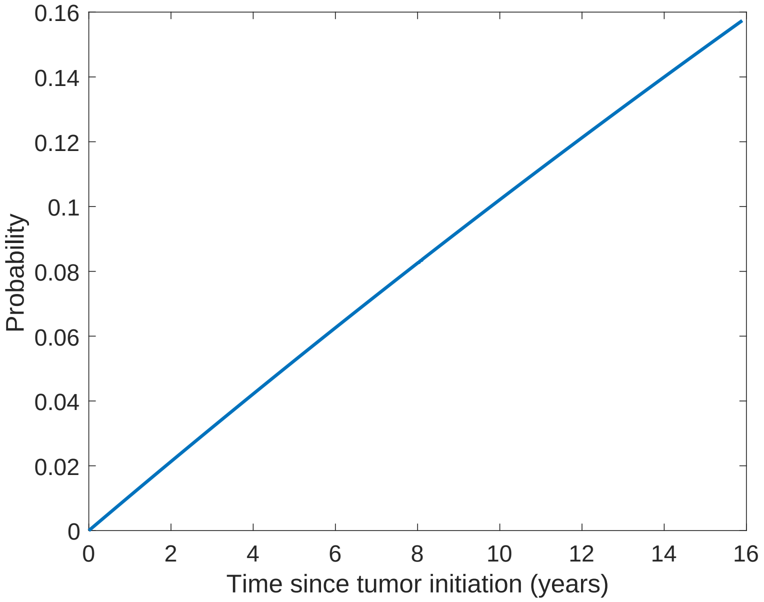

Figure S4: Probability of developing any combination of metastasis within a certain tumor age.

S3 Supplementary tables

Table S1: Dissemination rates. Values maximizing the likelihood of the whole data set and different subtype groups respectively. 95% confidence intervals in brackets.

	Parameter			
Subtype	*tubo*	*tulu*	*tuli*	*tubr*
All	0.00618<br>(0.00601-0.00629)	0.00269<br>(0.00261-0.00276)	0.00158<br>(0.00152-0.00163)	0.000321<br>(0.000293-0.000344)
HoR-/HER2+	0.00756<br>(0.00692-0.00801)	0.0062<br>(0.00552-0.00666)	0.00566<br>(0.00507-0.00616)	0.000689<br>(0.000527-0.000857)
HoR+/HER2+	0.0094<br>(0.00881 0.00980)	0.00427<br>(0.00395 0.00451)	0.00375<br>(0.00346-0.00403)	0.00054<br>(0.000421-0.000633)
HoR+/HER2-	0.00592<br>(0.00578-0.00603)	0.00201<br>(0.00193-0.00207)	0.00095<br>(0.00088-0.00100)	0.000226<br>(0.000195-0.000252)
HoR-/HER2-	0.00433<br>(0.00400-0.00458)	0.00395<br>(0.00367-0.00417)	0.00191<br>(0.00172-0.00207)	0.000559<br>(0.000461-0.000658)

	Parameter	
Subtype	*luli*	*lubr*
All	0.126<br>(0.116-0.133)	0.0404<br>(0.0364-0.0444)
HoR-/HER2+	0.164<br>(0.126-0.190)	0.0611<br>(0.0446-0.0742)
HoR+/HER2+	0.176<br>(0.148-0.201)	0.0350<br>(0.0244-0.0442)
HoR+/HER2-	0.106<br>(0.094-0.115)	0.0327<br>(0.0268-0.0379)
HoR-/HER2-	0.118<br>(0.097-0.135)	0.0562<br>(0.0427-0.0650)

Table S2: Mean absolute percentage error (MAPE) between data and model. The number of patients in respective group is shown in parenthesis. Average MAPE for each subtype group displayed in the the right column, while average MAPE for each metastasis type is displayed in the last row.

	Metastasis type (No.)				
Subtype (No.)	Bone (7094)	Lung (3109)	Liver (2649)	Brain (677)	Average
All (317166)	48.7	61.6	72.4	22.5	51.3
HoR-/HER2+ (13406)	42.7	48.2	52.8	11.3	38.8
HoR+/HER2+ (32504)	45.6	40.0	31.1	11.1	32.0
HoR+/HER2- (235828)	50.8	60.7	73.5	31.2	54.16
HoR-/HER2- (35428)	27.4	31.6	46.1	9.0	28.5
Average	43.0	48.4	55.2	17.0	

Table S3: Ratio of primary and secondary dissemination rates. Primary dissemination represented by *tuli* and *tubr* and secondary dissemination represented by *luli* and *lubr*. Errors in brackets are estimated from minimum and maximum possible ratios taking into account 95 % confidence intervals of dissemination rates.

	Ratios	
Subtype	*luli*:*tuli*	*lubr*:*tubr*
All	79.7 (71.2-87.5)	126 (106-152)
HoR-/HER2+	29.0 (20.5-37.5)	88.7 (52.0-140)
HoR+/HER2+	46.9 (36.7-58.1)	65 (38.5-105)
HoR+/HER2-	112 (94.3-131)	145 (106-194)
HoR-/HER2-	62 (46.7-78.5)	101 (64.9-141)

Supplementary material references

[S50] Sten Friberg and Stefan Mattson. On the growth rates of human malignant tumors: implications for medical decision making. *Journal of surgical oncology*, 65(4):284–297, 1997.

[S51] Socorro María Rodríguez-Pinilla, David Sarrió, Emiliano Honrado, David Hardisson, Francisco Calero, Javier Benitez, and José Palacios. Prognostic significance of basal-like phenotype and fascin expression in node-negative invasive breast carcinomas. *Clinical cancer research*, 12(5):1533–1539, 2006.

[S52] Hagen Kennecke, Rinat Yerushalmi, Ryan Woods, Maggie Chon U Cheang, David Voduc, Caroline H Speers, Torsten O Nielsen, and Karen Gelmon. Metastatic behavior of breast cancer subtypes. *Journal of clinical oncology*, 28(20):3271–3277, 2010.

[S53] Shi Wei and Gene P Siegal. Surviving at a distant site: The organotropism of metastatic breast cancer. In *Seminars in diagnostic pathology*, volume 35, pages 108–111. Elsevier, 2018.

[S54] Florian Heitz, Philipp Harter, Hans-Joachim Lueck, Annette Fissler-Eckhoff, Fatemeh Lorenz-Salehi, Stefanie Scheil-Bertram, Alexander Traut, and Andreas du Bois. Triple-negative and her2-overexpressing breast cancers exhibit an elevated risk and an earlier occurrence of cerebral metastases. *European journal of cancer*, 45(16):2792–2798, 2009.

[S55] R Largillier, J-M Ferrero, J Doyen, J Barriere, M Namer, V Mari, A Courdi, JM Hannoun-Levi, F Ettore, I Birtwisle-Peyrottes, et al. Prognostic factors in 1038 women with metastatic breast cancer. *Annals of Oncology*, 19(12):2012–2019, 2008.
